# Crystal, spectroscopic and quantum mechanics studies of Schiff bases derived from 4-nitrocinnamaldehyde

**DOI:** 10.1038/s41598-021-87370-0

**Published:** 2021-04-14

**Authors:** Friday E. Ani, Collins U. Ibeji, Nnamdi L. Obasi, Monsuru T. Kelani, Kingsley Ukogu, Gideon F. Tolufashe, Segun A. Ogundare, Oluwatoba E. Oyeneyin, Glenn E. M. Maguire, Hendrik G. Kruger

**Affiliations:** 1 Department of Pure and Industrial Chemistry, Faculty of Physical Sciences, University of Nigeria, Nsukka, 410001 Enugu State Nigeria; 2 Catalysis and Peptide Research Unit, School of Health Sciences, University of KwaZulu-Natal, Durban, 4041 South Africa; 3 Department of Chemical Sciences, Olabisi Onabanjo University, P. M. B. 2002, Ago-Iwoye, Nigeria; 4 School of Chemistry and Physics, University of KwaZulu-Natal, Durban, 4041 South Africa; 5 Department of Chemistry and Biochemistry, Faculty of Sciences, University of Porto, 4169-007 Porto, Portugal; 6 Department of Chemical Sciences, Adekunle Ajasin University, Akungba-Akoko, Ondo State Nigeria

**Keywords:** Computational chemistry, Chemistry, Physical chemistry

## Abstract

Two Schiff bases, (E)-1-(4-methoxyphenyl)-N-((E)-3-(4-nitrophenyl)allylidene)methanamine (compound **1)** and (E)-N-((E)-3-(4-nitrophenyl)allylidene)-2-phenylethanamine (compound **2)** have been synthesized and characterized using spectroscopic methods; time of flight MS, ^1^H and ^13^C NMR, FT-IR, UV–VIS, photoluminescence and crystallographic methods. The structural and electronic properties of compounds **1** and **2** in the ground state were also examined using the DFT/B3LYP functional and 6-31 + G(d,p) basis set, while the electronic transitions for excited state calculations were carried out using the TD-DFT/6-31 + G(d,p) method. The Schiff base compounds, **1** and **2** crystallized in a monoclinic crystal system and the P2_1_/c space group. The emission spectra of the compounds are attributed to conjugated π-bond interaction while the influence of the intra-ligand charge transfer resulted in a broad shoulder for **1** and a double emission peak for **2**. The calculated transitions at 450 and 369 nm for **1** and **2** respectively are in reasonable agreement with the experimental results. The higher values of dipole moment, linear polarizability and first hyperpolarizability of **1**, suggest a better optical property and better candidate for the development of nonlinear optical (NLO) materials.

## Introduction

Schiff bases are derived from the condensation reaction between primary amines and aldehydes or ketones and was first described by Hugo Schiff^1^. Schiff bases as ligands have been extensively studied, essentially due to their broad applications in several fields such as catalysis^2,3^, solid phase extraction^4^, synthesis^5^, antibacterial^6^, anti-inflammatory^7^ and antitumor agents^8,9^. The all-embracing applications of Schiff bases are possible due to the flexibility in the synthesis route, coordination ability to metal centre, structure, and presence of an imine functional group (–N=CH–)^10–12^. They have been reported to possess electrochemical and optical properties in sensor devices^13^. Schiff bases can also stabilize several metals in many oxidation states, which controls the activity of metals in great variation of catalytic conversions^14^. They coordinate with metals through the imine N atom^15–17^. Schiff bases also display a wide range of industrial application which includes their use as dyes and pigments^18–20^. These compounds show photochromic and thermochromic properties in the solid state^21–23^ and have also been reported for their use as optical limiters in laser equipment^24,25^. Organic molecules of the Donor–Acceptor (D-A) type molecules have been investigated and reported for their optical limiting applications which are necessary to protect sensors from dangerous laser beams^26^. They are especially considered because the π-electron movement from the donor part to the acceptor part results in larger NLO susceptibilities^27^. With the aid of computational techniques, it has become possible to predict the physico-chemical properties of chemical and biological systems^28^. Density functional theory (DFT)^29^ has been proven to be a viable method of determining many molecular properties such as geometry, dipole moment, vibrational frequency, etc. with high accuracy at a reasonable cost^30,31^. Nonlinear optical properties of organic systems with donor–acceptor (D–A) configuration have been successfully predicted with DFT^27,32,33^. Herein, 4-nitrocinnamaldehyde is employed to study the impact of the extent of π-conjugation on the electronic properties of the resulting Schiff bases due to the electron-withdrawing nitro (–NO_2_) group. To the best of our knowledge, scanty work exists regarding Schiff bases derived from 4-nitrocinnamaldehyde. Hence, two novel Schiff bases, ((E)-1-(4-methoxyphenyl)-N-((E)-3-(4-nitrophenyl)allylidene)methanamine and (E)-N-((E)-3-(4-nitrophenyl)allylidene)-2-phenylethanamine) were synthesized and characterized. The Photoluminescence and non-linear optical properties of the compounds were also investigated experimentally and computationally. This study, therefore, will provide insight into the optical applications of this class of Schiff bases derived from 4-nitrocinnamaldehyde.

## Experimental

### Reagents and apparatus

The chemicals used were of analytical grade and they were used without further purification. The IR spectra were recorded with an FT-IR spectrometer (Perkin Elmer, USA). Photoluminescence spectra were obtained with an LS55 Fluorescence Spectrometer (Perkin Elmer, UK). The NMR spectra were collected and recorded on a Bruker AVANCE III 400 MHz spectrometer using Topspin 3.1 (Bruker, Karlsruhe, Germany). The chemical shifts were referenced to the solvent peak, CDCl_3_, at δ = 7.26 ppm and δ = 77.16 ppm for ^1^H and ^13^C spectra, while TMS was used as the internal standard. The 2D NMR spectra were also collected and recorded on the same NMR instrument. Data of the single crystal were collected on a Bruker SMART APEX2 area detector diffractometer. All the synthesis and analysis were carried out at Catalysis and Peptide Research Unit, School of Health Sciences and School of Chemistry and Physics, University of KwaZulu-Natal, Durban South Africa.

### Synthesis of the Schiff base compounds

#### Synthesis of (E)-1-(4-methoxyphenyl)-N-((E)-3-(4-nitrophenyl)allylidene)methanamine (1)

**Scheme 1:**
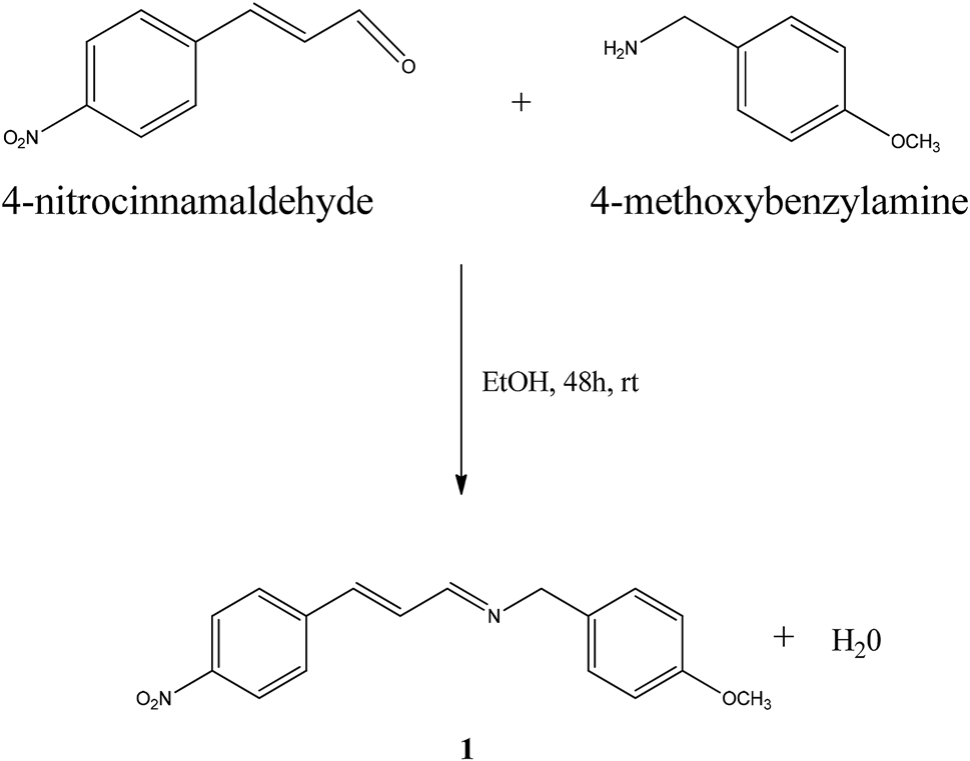
Synthesis of (E)-1-(4-methoxyphenyl)-N-((E)-3-(4 nitrophenyl)allylidene)methanamine (**1**).

4-Methoxybenzylamine (230 mg; 1.70 mmol) was added to a solution of 4-nitrocinnamaldehyde (300 mg; 1.70 mmol) in ethanol (15 ml). The mixture was stirred for 48 h in a 100 ml round-bottom flask at room temperature. The resulting precipitates were filtered and washed with cold ethanol and then dissolved in hot ethanol and allowed to stand. Single crystals of the compound **1** (Scheme 1), suitable for X-ray diffraction study, were obtained as the ethanol evaporated slowly. The following properties are obtained: ((C_17_H_16_N_2_O_3_). Yield: 89%, colour: orange, m.p.: 94 °C, MS-TOF (m/z): [M + H]^+^ calculated for C_17_H_16_N_2_O_3_: 297.123; found: 297.124 (Figure S1). See Table 4 and Table S1 for other spectroscopic data.

#### Synthesis of (E)-N-((E)-3-(4-nitrophenyl)allylidene)-2-phenylethanamine (2)

**Scheme 2:**
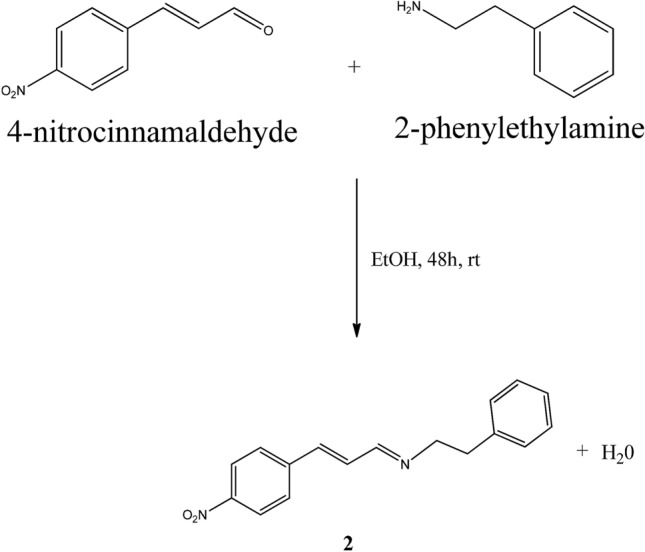
Synthesis of (E)-N-((E)-3-(4-nitrophenyl)allylidene)-2-phenylethanamine (**2**).

Compound **2** (Scheme 2) was synthesized under the same experimental condition as **1**. The following properties are obtained: (C_17_H_16_N_2_O_2_). Yield: 87%, colour: orange, m.p.: 95–96 °C MS-TOF (m/z): [M + H]^+^ calculated for C_17_H_16_N_2_O_2_: 281.128; found: 281.129. See Table 4 and Table S1 for other spectroscopic data.

### X-ray determination of the compounds

Single rod-shaped crystals of **1** and single plank-shaped crystals of **2** were obtained by recrystallization from ethanol and methanol respectively. Suitable crystals had dimensions of 0.38 × 0.22 × 0.14 mm^3^ for compound **1** and 0.32 × 0.24 × 0.13 mm^3^ for **2**. These were selected and mounted on a suitable support on the single crystal X-ray instrument. The crystals were kept at a steady temperature (*t* = 100(2) k) during data collection. The structures were resolved with the shelxs-2013^34^ structure solution program using the intrinsic phasing solution method and by using olex2^34^ as the graphical interface. The models were refined with version 2016/6 of shelxl^35^ using least squares minimisation. CCDC-1998746 and CCDC-1998749 contain the supplementary crystallographic data information for **1** and **2** respectively. This data can be obtained freely from the Cambridge Crystallographic Data Centre through www.ccdc.cam.ac.uk/data_request/cif. The crystal data and details of the structure solution and refinement are given in Table 1, bond lengths and angles are given in Tables 2 and 3.

**Table 1:** Crystallographic data of the Schiff base compounds.

Compound	**1**	**2**
Formula	C_17_H_16_N_2_O_3_	C_17_H_16_N_2_O_2_
*D*_*calc.*_/ g cm^−3^	1.368	1.277
Abs. coef./mm^−1^	0.095	0.085
Formula Weight	296.32	280.32
Colour	Orange	Orange
Shape	Rod	Plank
Size/mm^3^	0.38 × 0.22 × 0.14	0.32 × 0.24 × 0.13
*T*/K	100(2)	100(2)
Crystal System	Monoclinic	Monoclinic
Space Group	*P*2_1_/*c*	*P*2_1_/*c*
*a*/Å	13.8304(2)	8.0465(2)
*b*/Å	13.1877(2)	6.25250(10)
*c*/Å	8.20150(10)	29.0701(6)
α/°	90	90
β/°	105.8830(10)	94.5220(10)
γ/°	90	90
V/Å^3^	1438.77(4)	1457.99(5)
*Z*	4	4
*Z'*	1	1
Wavelength/Å	0.71073	0.71073
Radiation type	MoK□	MoK□
*θ*_*min*_/°	2.175	2.539
*θ*_*max*_/°	28.306	28.611
Measured Refl	18,648	20,491
Independent Refl	3505	3710
Reflections with I > 2(I)	2802	3242
*R*_*int*_	0.0286	0.0163
Parameters	200	190
Restraints	0	0
Largest Peak	0.318	0.359
Deepest Hole	−0.192	−0.220
GooF	1.026	1.034
*wR*_*2*_ (all data)	0.0991	0.0999
*wR*_*2*_	0.0894	0.0950
*R*_*1*_ (all data)	0.0501	0.0430
*R*_*1*_	0.0363	0.0368

**Table 2:** Selected Experimental and calculated bond lengths (Ǻ) of **1** and **2**.

**1**			**2**		
Atoms	Experimental	Computed	Bond length (Ǻ)	Experimental	Computed
O1–N1	1.2266(13)	1.23131	O1–N1	1.2297(12)	1.23157
N2–C9	1.2713(15)	1.27904	O2–N1	1.2257(12)	1.23180
N2–C10	1.4631(15)	1.45517	C16–C15	1.3857(16)	1.39596
O3–C14	1.3705(14)	1.36708	C16–C17	1.3861(15)	1.39488
O3–C17	1.4348(14)	1.41815	C15–C14	1.3849(16)	1.39551
O2–N1	1.2258(13)	1.23131	C14–C13	1.3896(15)	1.39543
N1–C1	1.4660(15)	1.46908	C13–C12	1.3927(14)	1.40109
C5–C6	1.3807(16)	1.38930	C12–C11	1.5085(14)	1.38653
C5–C4	1.4060(15)	1.40906	C12–C17	1.3937(14)	1.40181
C9–C8	1.4515(16)	1.46342	C11–C10	1.5244(14)	1.54432

**Table 3:** Selected experimental and calculated bond angles (°) of **1** and **2**.

**1**			**2**		
Atoms	Experimental	Computed	Atoms	Experimental	Computed
C9–N2–C10	115.87(10)	122.09321	C15–C16–C17	120.24(10)	120.11142
C14–O3–C17	116.77(9)	118.11922	C14–C15–C16	119.70(10)	119.56012
O1–N1–C1	118.48(10)	117.65622	C15–C14–C13	119.98(10)	120.10025
O2–N1–O1	123.43(10)	124.69859	C14–C13–C12	120.88(10)	120.10645
O2–N1–C1	118.08(9)	117.64519	C13–C12–C11	121.41(9)	120.95616
C6–C5–C4	120.65(11)	121.45528	C13–C12–C17	118.44(9)	118.32992
N2–C9–C8	122.81(11)	130.10955	C17–C12–C11	120.13(9)	120.69158

### Computational methods

Molecular chemical stability is a measure of the difference in HOMO and LUMO energies^36^. This energy gap between HOMO and LUMO can be used to determine molecular electrical transport properties. The electrophilicity and electronegativity index, chemical hardness and softness of a molecule were calculated using the HOMO and LUMO energy values as follows^37^:

1 $$\eta \approx \frac{I - A}{2}\, \left( {{\text{Chemical}}\,{\text{ hardness}}} \right)$$

2 $$\mu \approx - \chi = - \frac{I + A}{2}\, \left( {{\text{Electronegativity}}} \right)$$

3 $${\uppsi } = \frac{{{\upmu }^{2} }}{{2{\upeta }}}\, \left( {{\text{Electrophilicity}}\,{\text{ index}}} \right)$$

4 $${\upzeta } = { }\frac{1}{{2{\upeta }}}\, \left( {{\text{Softness}}} \right)$$

where A is the electron affinity and I, the ionization potential. A = − E_LUMO_, I = − E_HOMO_^38–41^. Thermodynamic energy parameters of compounds were also calculated using B3LYP/6-31G(d,p) method. The total static dipole moment (µ) and the average linear polarizability (${\overline{\upalpha }}$) for the Schiff base compounds were calculated using the B3LYP/6-31G(d,p) method (Eqs. 5–7), the first hyperpolarizability (β) were calculated using the Kleinmann’s symmetry^42,43^. β value is a measure of the second harmonic generation efficiency^44^:

5 $$\mu = \left( {\mu_{x}^{2} + \mu_{y}^{2} + \mu_{y}^{2} } \right)^{{{\raise0.7ex\hbox{$1$} \!\mathord{\left/ {\vphantom {1 2}}\right.\kern-\nulldelimiterspace} \!\lower0.7ex\hbox{$2$}}}}$$

6 $${\overline{\upalpha }} = \frac{1}{3}\left( {\alpha_{xx} + \alpha_{yy} + \alpha_{zz} } \right)$$

7 $$\beta = \left[ {\left( {\beta_{xxx} + \beta_{xyy} + \beta_{xzz} } \right)^{2} + \left( {\beta_{yyy} + \beta_{xxy} + \beta_{yzz} } \right)^{2} + \left( {\beta_{zzz} + \beta_{xxz} + \beta_{yyz} } \right)^{2} } \right]^{{{\raise0.7ex\hbox{$1$} \!\mathord{\left/ {\vphantom {1 2}}\right.\kern-\nulldelimiterspace} \!\lower0.7ex\hbox{$2$}}}}$$

### Natural bond orbital (NBO)

NBO analysis is an effective approach to understand the intra and intermolecular bonding and interaction among bonds, and information of charge transfer or conjugative interactions in molecular system^45,46^. The associated electron donor orbital, acceptor orbital and the interacting stabilization energy were derived from the second-order micro-disturbance theory. For each donor (*i*) and acceptor (*j*), the stabilization energy *E*^(*2*)^ associated with delocalization is approximated as^47^:

8 $$E^{\left( 2 \right) } = \Delta E_{ij} = q_{j} \frac{{F\left( {i,j} \right)^{2} }}{{\varepsilon_{j} - \varepsilon_{i} }}$$

where $q_{j}$ is the donor orbital occupancy, $\varepsilon_{i}$ and $\varepsilon_{j}$ are diagonal matrix elements and $F\left( {i,j} \right)$ is the off-diagonal Fock matrix element. B3LYP functional with 6-31 + G(d,p) basis was used for NBO calculation. All calculations were carried out using Gaussian16^48^ with the default convergence criteria, without any constraint on the geometry.

## Results and discussion

### Crystallographic study

**Figure 1:**
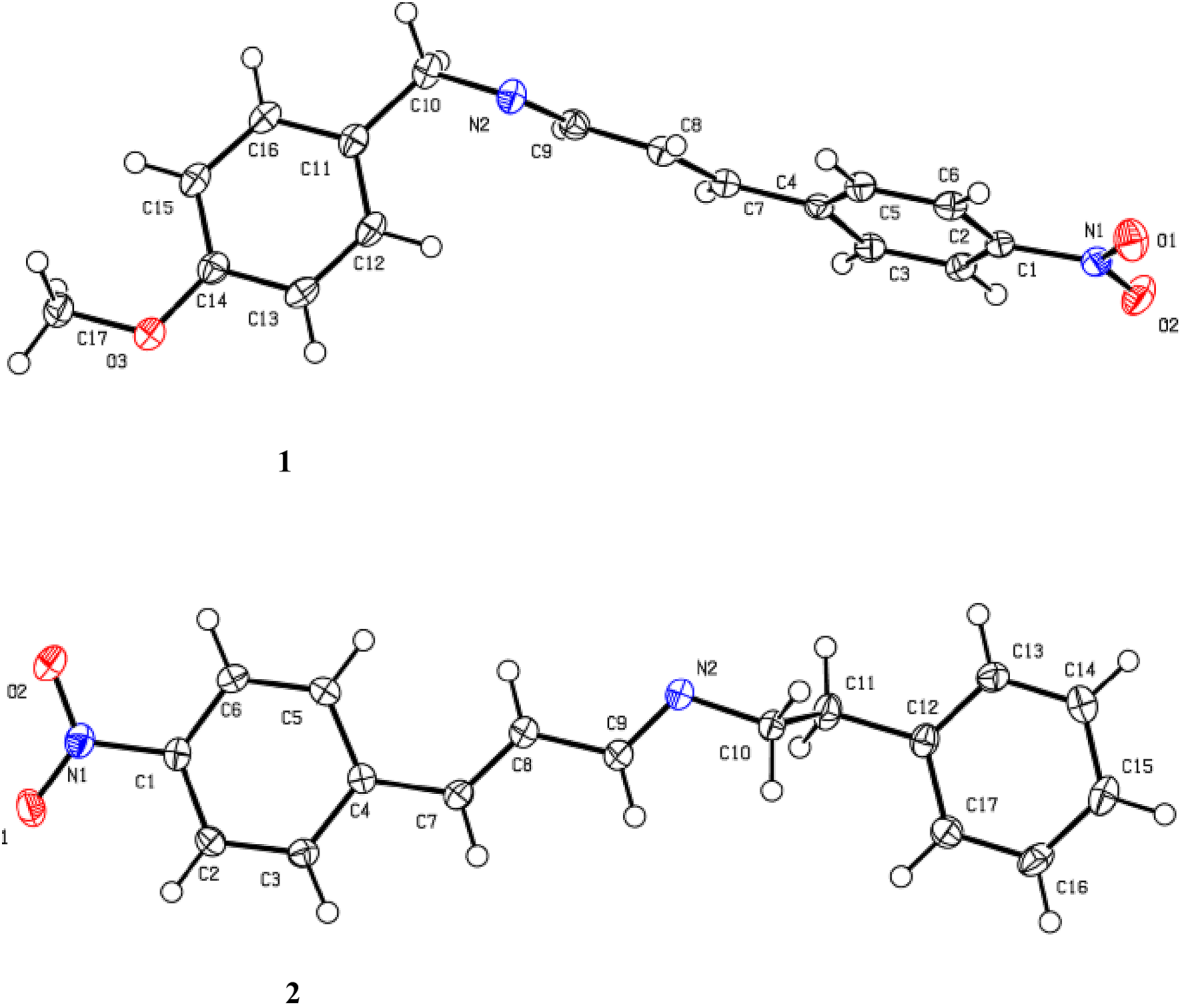
Crystal structures of the compounds with atom labelling (thermal ellipsoid, 50%).

The two compounds crystalized in the monoclinic crystal system and P2_1_/c space group, the ORTEP view of **1** and **2** are shown in Fig. 1. The crystal data and refinement details for the compounds are presented in Table 1. The compounds lie on centre of symmetry. The bond lengths in the compounds both experimental and computed are within the expected range. From the data in Tables 2 and 3, the bond distance of equivalent atoms in the compounds are equal, the C7=C8 length is 1.3383 (16)Å (1.34949 Å) which falls within the average C=C double bond length (1.34 Å). The C8–C9 bond length is (1.46342 Å) which is also in good agreement with the C–C single bond length (1.48 Å)^49^. Also, the N=C bond length (1.2713(15) Å) (1.27904 Å) agrees quite well with the reported value 1.279 Å^50^ (Table 2). The dihedral angles of the atoms (C10–N2–C9–C8) in **1** and **2** are found to be 178.70° and 179.50° respectively (Table 3).

### Infrared spectra

The FT-IR spectra of the compounds **1** and **2** are presented in Figure S2 and S3. The strong peaks at 1593 and 1591 cm^−1^ for **1 and 2** correspondingly are assigned to the azomethine ѵ(CH=N) vibrations which indicate the formation of the Schiff bases. The peaks around 3088 (**1**) and 3031 (**2**) cm^−1^ are diagnostics of aromatic C–H vibrational stretching frequency^51^. While the bands at 2947 (**1**) and 2926 (**2**) cm^−1^ are due to aliphatic C–H stretching vibrations in the respective compounds. The peak at 1242 cm^−1^ in **1** spectrum is assigned to C–O stretching vibration but absent in the spectrum of **2.** While those at 1341 and 1443 cm^−1^ in **1**, and 1347 and 1506 cm^−1^ in **2** spectrum are assigned to the –NO_2_ symmetric and antisymmetric stretching vibrational frequency respectively^52^. The FT-IR assignments for the compounds are presented in Table 4.

**Table 4:** FT-IR Spectral assignments.

**1**	**2**	Assignments^a^
3088	3031	υ_aromatic_(C–H)
2947	2926	υ_aliphatic_(C–H)
1593	1591	υ(C=N)
1443, 1341	1506, 1347	υ(NO_2_)
1242, 1040		υ_methoxy_(C–O)
1106	1106	γ_aromatic_(C–H)
816, 734, 690	974, 836, 752, 696	ζ_aromatic_(C–H)

^a^ υ: stretching, γ: bending, ζ: out of plane bending.

### Electronic spectra

The UV–VIS electronic absorption spectra recorded in chloroform for compounds **1** and **2** are presented in Figure S4–S5. A broad absorption band in the range 250–370 nm with peaks at 322 and 320 nm corresponding to 3.86 and 3.89 eV for **1** and **2** respectively are associated with π–π* transitions in the compounds^53^. The calculated energy gap for **1** and **2** was 3.73 and 3.70 eV respectively. However, the molar absorptivity (ε) of **1** (1.1 × 10^6^ M^−1^ cm^−1^) was greater than that of **2** (3.2 × 10^5^ M^−1^ cm^−1^); an indication of the influence of the electron donating effect of the *p*-methoxy substituent in **1**.

### ^1^H and ^13^C NMR spectroscopic study

The ^1^H NMR, ^13^C NMR and the 2D spectra for **1** and **2** are shown in Figures S6–S10 in the supporting document. For compound **1**, the proton resonance at 3.79 ppm was assigned to the methoxy protons and labelled as H-17 (Table S1). This exhibits HMBC correlation only to a quaternary carbon (158.87 ppm) assigned to C-14. HMBC interactions occur between the C-14 and proton signals recorded at 7.21 and 6.89 ppm, which could be assigned to H-12/16 and H-13/15, respectively or vice versa. The signal at 4.69 ppm labelled H-10 shows HMBC interactions with a quaternary and a methine carbons recorded at 130.77 and 129.34 ppm, respectively, while the proton resonating at 7.21 ppm was assigned H-12/16. By elimination, the signals at 6.89 ppm and 130.77 ppm were attributed to H-13/15 and C-11. In addition, H-10 displays an HMBC correlation to a carbon registered at 161.97 ppm (which corresponds to a proton at 8.14 ppm) and a COSY correlation to a signal at 7.06 ppm (7.60 Hz). This signal in turn displays a COSY interaction to another signal at 7.00 ppm with a *trans*-coupling constant of 16 Hz. Hence, the resonance at 7.06 ppm was assigned to H-2 and the one at 8.14 ppm was attributed to H-1. Two resonances registered at 7.59 and 8.21 ppm exhibiting COSY interactions with an *ortho*-coupling constant of 8.80 Hz, could be assigned as H-5/9 and H-6/8 respectively, or vice versa. Furthermore, the signal at 7.00 ppm displays an HMBC interaction with a carbon signal at 127.71 ppm corresponding to the proton at 7.59 ppm which was assigned H-5/9, while the signal at 8.21 ppm was assigned H-6/8. Hence, by elimination, the signal at 7.00 ppm was assigned to H-3. All quaternary carbons and methylene carbons were differentiated from methine and methyl carbons with the aid of ^13^C attached proton test (APT) experiment. The methoxy proton resonance recorded at 3.79 ppm in **1** serves as a diagnostic structural difference between the two compounds. This observation is corroborated by the presence of C-O stretching band at 1242 cm^−1^ in compound **1**, but absent in **2**. The NMR spectra of compound **2** was elucidated similarly, the discussion is presented in the Supplementary Material.

### Photoluminescence (spectroscopic analysis)

The photoluminescence study of **1** and **2** was carried out in chloroform at room temperature. The excitation and emission spectra of the compounds **1** and **2** as seen in Figures S11 and S12 showed that the excitation bands were in the UV region at 302 nm with a shoulder at 345 nm in **1**, and at 345 nm with a shoulder at 360 nm in **2**. However, the emission bands were in the visible region centred at 486 nm with a shoulder at 446 nm in **1**, while a double peak was observed at 455 and 478 nm in **2**. The main band of the excitation spectra could be associated with π–π* intra-ligand transition and the shoulder suggested intra-ligand charge transfer band. The emission spectra of the compounds are attributed to conjugated π-bond interaction while the influence of the intra-ligand charge transfer resulted in a broad shoulder in **1** and a double emission peak in **2**^27,51^.

Electronic excited state calculations obtained using TD-DFT in chloroform as a solvent phase are presented in Table 5. Since **1** and **2** are conjugated systems with π bonds and aromatic rings, thus, it allows π–π* transitions in the UV–vis region with high extinction coefficients. The calculated transitions at 450 and 369 nm for **1** and **2** respectively agree reasonably with the experimental results.

**Table 5:** Electronic absorption spectral data of **1** and **2** molecules.

	Major contribution^a^	Wavelength (nm)		Oscillator strength (f)	Energy (eV)
		Calc	Expt		
**1**	H → L(99%)	450	486	0.0067	2.7570
	H-1 → L(99%)	359		0.9242	3.4539
	H-2 → L(47%)	340		0.001	3.6414
**2**	H → L(98%)	369	478	0.6816	3.3557
	H-1 → L(73%)	344		0.2471	3.6003
	H-3 → L(48%)	339		0.0263	3.6577

^a^ H: HOMO, L: LUMO.

### Nonlinear optical parameters

From the results in Table 6, 1 has higher values for dipole moment, linear polarizability and first hyperpolarizability; suggesting that **1** has a higher tendency to interact with an external field. The reason for this could be that the presence of methoxy group (–O–CH_3_) in **1** induces more polar and multipolar components than in **2**, with the β values of both molecules being dominated by the β_xxx_ tensor component, this is indicative that there is delocalization of charges in the β_xxx_ direction (Table S2). The optical properties of **1** and **2** were compared with the properties of urea and _L_-leucine nitrate (Table 6). Urea is one of the prototypical molecules applied in the study of the NLO properties of molecular systems. It was observed that both compounds in this study are better NLO materials based on their dipole moment and β values. According to the magnitudes of the polarizability (α) and the first hyperpolarizability (β) values of the investigated Schiff compounds, they may be useful in the development of NLO materials.

**Table 6:** Calculated total dipole moments (µ), average linear polarizability (${\overline{\upalpha }}$) and the first hyperpolarizability (β) and for the Schiff base compounds.

Parameter	**1**	**2**	Urea	l-leucine nitrate
µ (D)	4.35	3.54	1.37^a^	4.58^c^
${\overline{\upalpha }}$(a.u)	− 141.76	− 136.84	–	
β (a.u)	773.73	250.15	–	
β (× 10^−30^ esu)	6.68	2.16	0.65^b^	6.13^d^

a^54^, b, c, d^55^.

### HOMO–LUMO analysis

**Figure 2:**
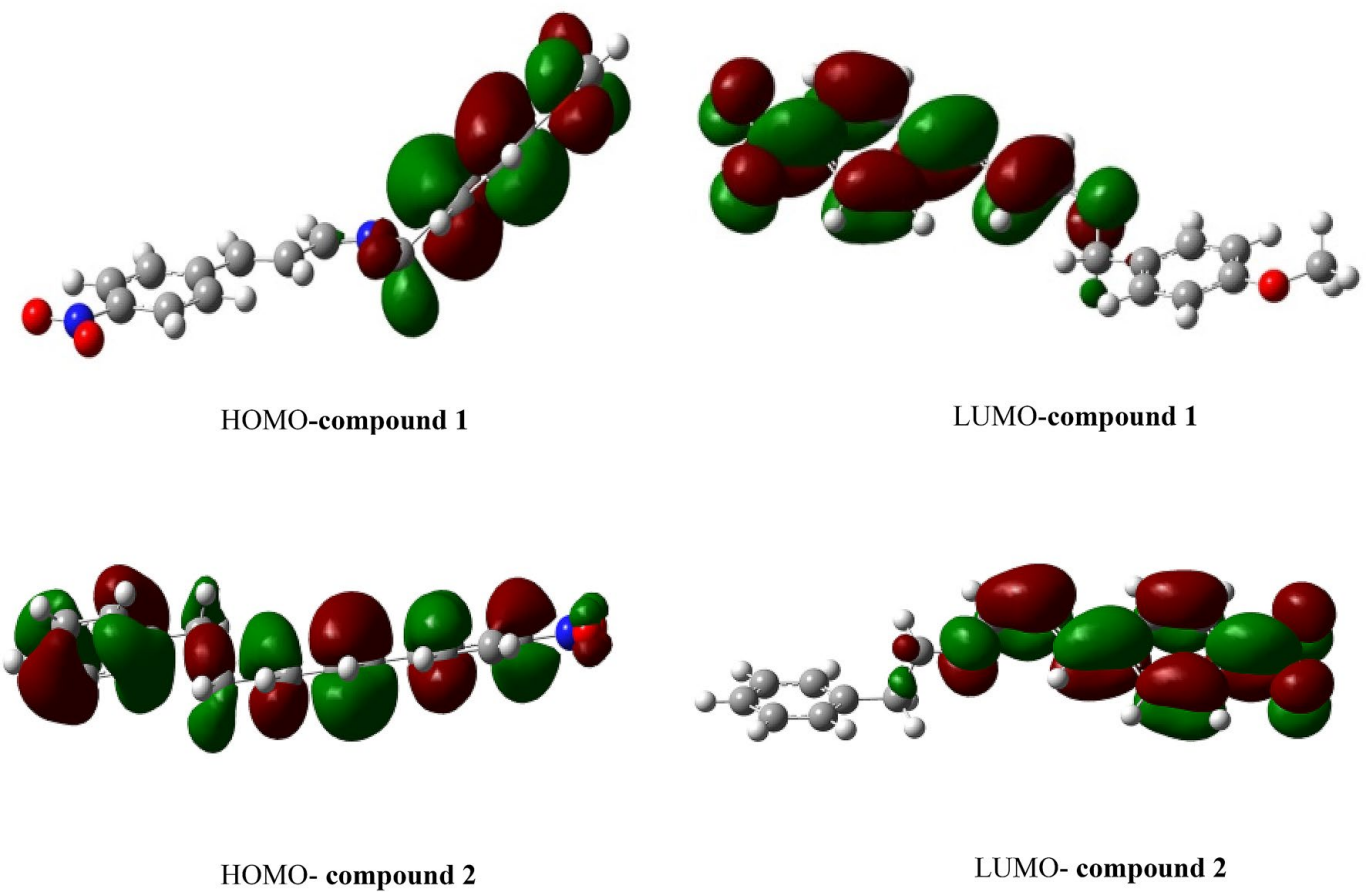
3D plots of HOMO and LUMO for the compounds.

The energy gap, chemical hardness, electrophilicity index, electronegativity and softness of the compounds are listed in Table 7. The energy gap of the one electron excitation from HOMO to LUMO for **1** and **2** are 2.76 and 3.57 eV, respectively. The lower energy gap of **1** indicates that it is more reactive and softer than **2**, the implication is that electrons are more easily migrated from the donor part to the acceptor part, it is also more polarizable and needs smaller energy for excitation than **2**. The 3D plots for the HOMO and LUMO of **1** and **2** are shown in Fig. 2. The variation of energy gap with dipole moment, polarizability and hyperpolarizability agrees with the expected trend. The lower the energy gap, the higher the dipole moment, polarizability and hyperpolarizability^26,56^. Analysis of the result showed that compound **1** has lower energy gap, higher dipole moment, higher polarizability and hyperpolarizability compared to **2**. Chemical hardness has a direct significance as it is derived from the energy gap, as seen in Eq. 1 while chemical softness increases as the energy gap reduces (Eq. 4). These parameters help in predicting excitation properties through electron transport. A soft molecule is characterized by low LUMO–HOMO gap which favours better chemical reactivity, and this is a measure of the polarizability and hyperpolarizability character of a compound^54^. **1** is termed a softer molecule with more chemical reactivity based on the descriptors indicating that higher electron transition occurs in 1 compared to **2.**

**Table 7:** The electronic properties and global reactivity descriptors of **1** and **2**.

Parameter (eV)	**1**	**2**
HOMO	− 6.06	− 6.81
LUMO	− 3.30	− 3.24
Energy gap	2.76	3.57
Chemical hardness (η)	1.38	1.79
Electronegativity (Ʋ)	4.68	5.03
Softness (ζ)	0.36	0.28

The thermodynamic parameters of 1 and 2 were obtained and listed in Table S3. Thermodynamics properties such as zero point energy (ZPE), enthalpy, Gibbs free energy (G^0^) are essential to establish the stability, structural and reactivity of systems^57,58^. The high values of G^0^ and ZPE (Table S3) suggests thermodynamic stability of compounds.

### Natural bond orbital (NBO) analysis

The larger the E^(2)^ value, the more intensive is the interaction between electron donors and electron acceptors, i.e. the more donating tendency from electron donors to electron acceptors and the greater the extent of conjugation of the entire system^59^. NBO calculations were performed on the molecules at the DFT/B3LYP/6-31G(d,p) level in order to explain the intra molecular rehybridization and delocalization of electron density within the studied molecules.

The second-order perturbation energies E^(2)^ associated with the delocalization of donor and acceptor bonds were presented in Table 8. The intramolecular hyperconjugative interactions formed by the orbital overlap between π*(C21–C25) and π*(C18–C19) bond orbitals of **1** coupled with a substituted methoxy at the gamma position resulted in a huge intramolecular charge transfer leading to stabilization of ∼217.77 kJ/mol of the system. With respect to **2**, as it has only a conjugated ring formed overlap between π(C27–C29) and π*(C32–C34) with a corresponding stabilization energy of 21.25 kJ/mol. Hydrogen bonding interactions was observed between the nitrogen lone pair and C–H antibonding orbital (LP (1)N33 → σ*C16–H36, LP(1)N18 → σ*C16–H21) with the energetic contribution (12.99 and 13.21 kJ/mol) for **1** and **2,** respectively. Electron intramolecular delocalization is obvious in the resonating of the nitrogen and oxygen lone pairs (LP (3) O13 → σ*N11 of both molecules given a close stabilization energy of 163.35 and 163.31 kJ/mol.

**Table 8:** The second-order perturbation energies E(2) associated with the delocalization of donor and acceptor bonds of **1** and **2** obtained at B3LYP/6-311G(d,p).

Donor (i)	Acceptor (j)	E^(2)a^ (kcal mol^−1^)
**1**		
BD (2) C_1_–C_2_	BD*(2) N_11_–O_12_	25.45
BD*(2) C_21_–C_25_	BD*(2) C_18_–C_19_	217.77
LP (2) O_12_	BD*(1) N_11_–O _13_	19.24
LP (3) O_13_	BD*(2) N_11_–O _12_	163.35
LP (1) N_33_	BD*(1) C_16_–H_36_	12.99
**2**		
BD (2) C_1_–C_2_	BD*(2) C_5_–C_6_	18.21
BD (2) C_27_–C_29_	BD*(2) C_32_–C_34_	21.25
LP (2) O_12_	BD*(1) N_11_–O_13_	19.24
LP (3) O_13_	BD*(2) N_11_–O_12_	163.31
LP (1) N_18_	BD*(1) C_16_–H_21_	13.21

a *E*^(2)^ means energy of hyperconjugative interactions (stabilization energy).

### Molecular electrostatic potential analysis

**Figure 3:**
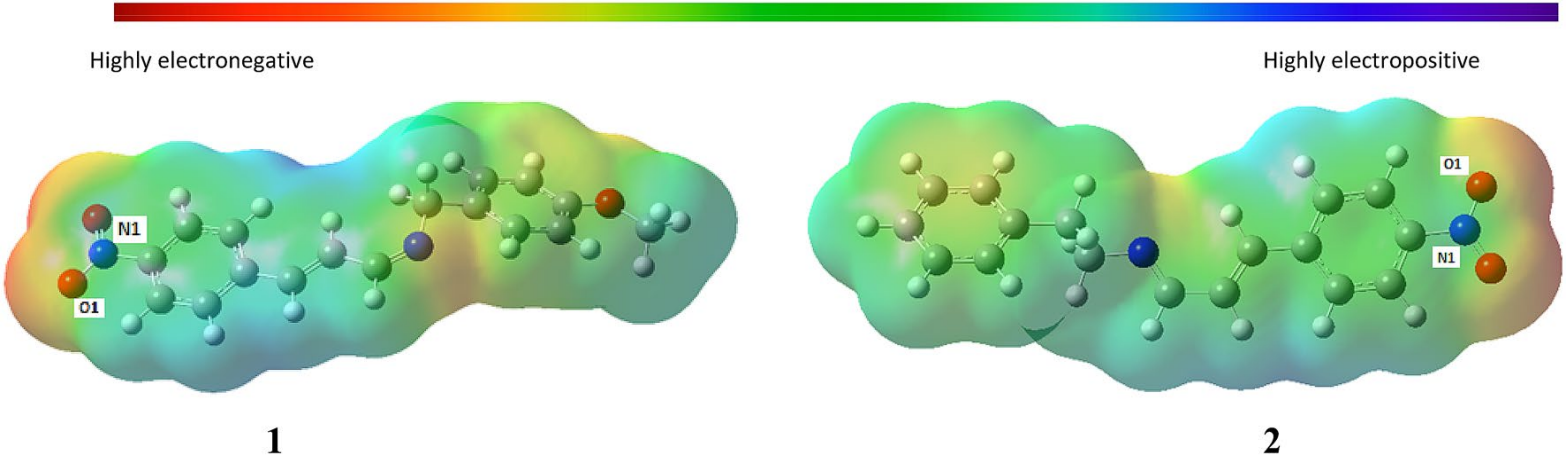
Molecular electrostatic potential (MEP) map of the compounds.

The molecular electrostatic potential (MEP), V(r) at a specified point r(x, y, z) within the environs of a molecule is given in terms of the interaction energy between a test positive charge (a proton) located at point r and an electrical energy which is generated from the molecule electrons and nuclei. The V(r) values for the studied systems are calculated by the Eq. (9)^60^

9 $$V\left(r\right)=\sum_{A}\frac{{Z}_{A}}{({R}_{A}-r)}-\int \frac{\rho ({r}^{^{\prime}})}{\left|{r}^{^{\prime}}-r\right|}d{r}^{^{\prime}}$$

where Z_A_ is the charge of nucleus A located at R_A_ in the vicinity of the system, ρ(r′) is the electronic density function of the molecule, and r′ is the dummy integration variable^61^. The MEP at the B3LYP/6-31G(d,p) optimized geometry of the compounds were calculated in order to predict the sites of nucleophilic and electrophilic attack on the Schiff base compounds. MEP is determined by electron density and is a reliable indicator of sites of possible nucleophilic and electrophilic attack as well as sites for hydrogen-bonding interaction on a compound. From the MEP map of the compounds as shown in Fig. 3, electrophilic and nucleophilic attack could likely take place on the O1 and N1 as they are the most electronegative and electropositive sites on the maps.

## Conclusion

Compounds of (E)-1-(4-methoxyphenyl)-N-((E)-3-(4-nitrophenyl)allylidene)methanamine and (E)-N-((E)-3-(4-nitrophenyl)allylidene)-2-phenylethanamine have been synthesized and characterized using ^1^H and ^13^C NMR, UV, FT-IR spectroscopy, Time of flight MS, X-ray crystallographic methods. The structural and photophysical properties of the studied systems were rationalized via DFT and TD-DFT calculations. The structural analysis from the crystallographic data shows that the compounds were obtained as monoclinic crystals. The structural difference between the two compounds were established from the NMR study and confirmed by the single crystal X-ray crystallographic data. Bond lengths and bond angles obtained experimentally closely agree with the theoretical values. Similarly, the energy gaps from the electronic spectra associated with π–π^*^ transitions in the compounds are comparable with those obtained theoretically. The photoluminescence properties of the compounds were investigated, and the emission spectra obtained are attributed to conjugated π-bond interaction characterised by high intramolecular charge transfer and leading to the stabilization of the studied systems. The low ∆E, high polarizability (α) and the first hyperpolarizability (β) values obtained in this study suggests that the studied compounds are good condidatesfor the development of NLO materials.

## Supplementary Information

Supplementary Information.
