# Supplementary material for: Crystal, spectroscopic and quantum mechanics studies of Schiff bases derived from 4-nitrocinnamaldehyde
Source: Sci Rep. 2021 Apr 14;11:8151. doi: 10.1038/s41598-021-87370-0 (PMC8046777; doi:10.1038/s41598-021-87370-0)
Supplement: Supplementary file 1 — Supplementary Information. [file 41598_2021_87370_MOESM1_ESM.pdf]

Crystal, Spectroscopic and Quantum mechanics studies of Schiff Bases derived from 4-nitrocinnamaldehyde

Friday E. Ani[1,2], Collins U. Ibeji [1,2*], Nnamdi L. Obasi[1*], Monsuru T. Kelani[2], Kingsley Ukogu[1], Gideon F. Tolufashe[2,5], Segun A. Ogundare[3,4], Oluwatoba E. Oyeneyin[6], Glenn E. M. Maguire,[2,4] and Hendrik G. Kruger.[2]

[1]Department of Pure and Industrial Chemistry, Faculty of Physical Sciences, University of Nigeria, Nsukka 410001, Enugu State, Nigeria.

[2]Catalysis and Peptide Research Unit, School of Health Sciences, University of KwaZulu-Natal, Durban 4041, South Africa.

[3]Department of Chemical Sciences, Olabisi Onabanjo University, P. M. B. 2002, Ago-Iwoye, Nigeria

[4]School of Chemistry and Physics, University of KwaZulu-Natal, Durban 4041, South Africa

[5]Department of Chemistry and Biochemistry, Faculty of Sciences, University of Porto, 4169-007 Porto, Portugal

[6]Department of Chemical Sciences, Adekunle Ajasin University, Akungba-Akoko, Ondo State, Nigeria.

*Corresponding author e-mails: ugochukwu.ibeji@unn.edu.ng, (ICU)nnamdi.obasi@unn.edu.ng (ONL).

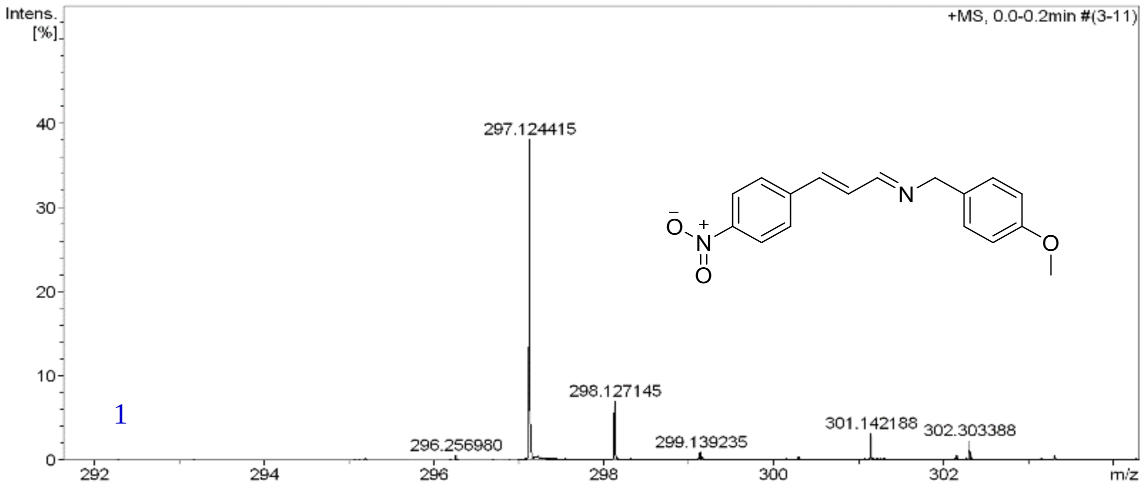

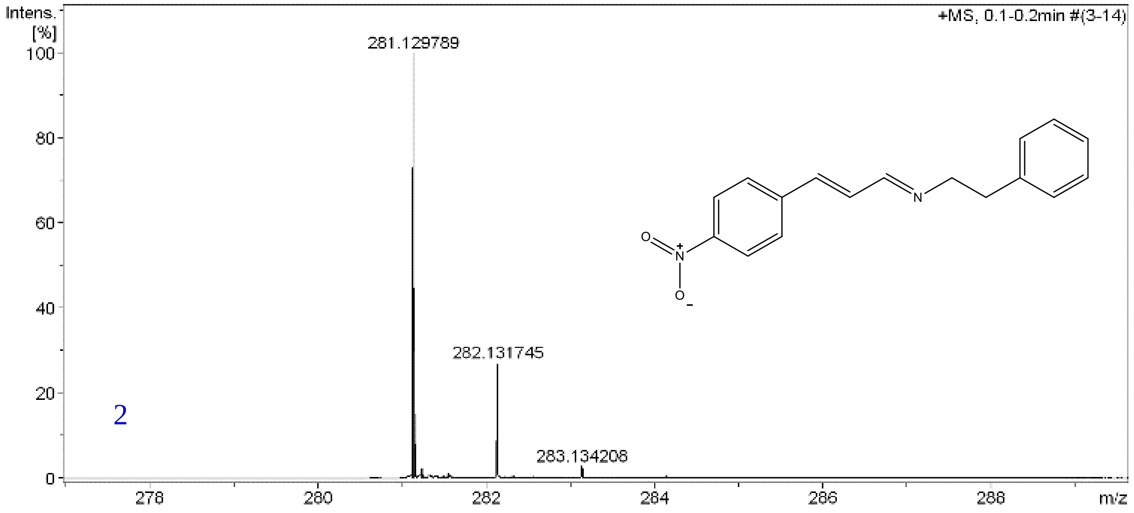

Figure S1: Time of flight mass spectra of the Schiff base compounds

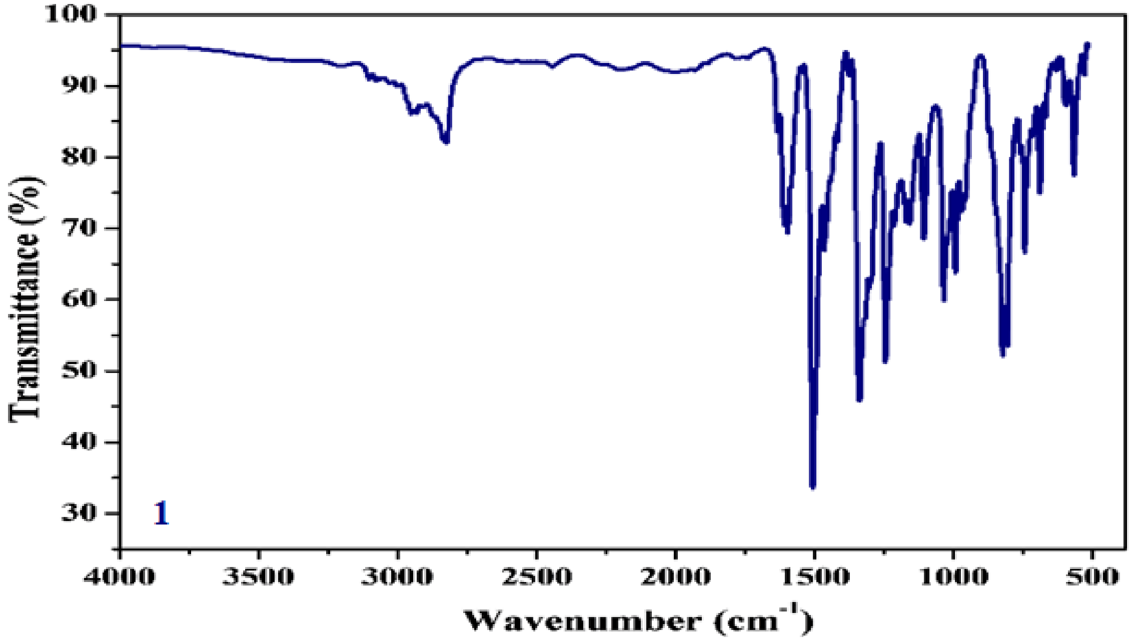

Figure S2: FT-IR spectrum of 1

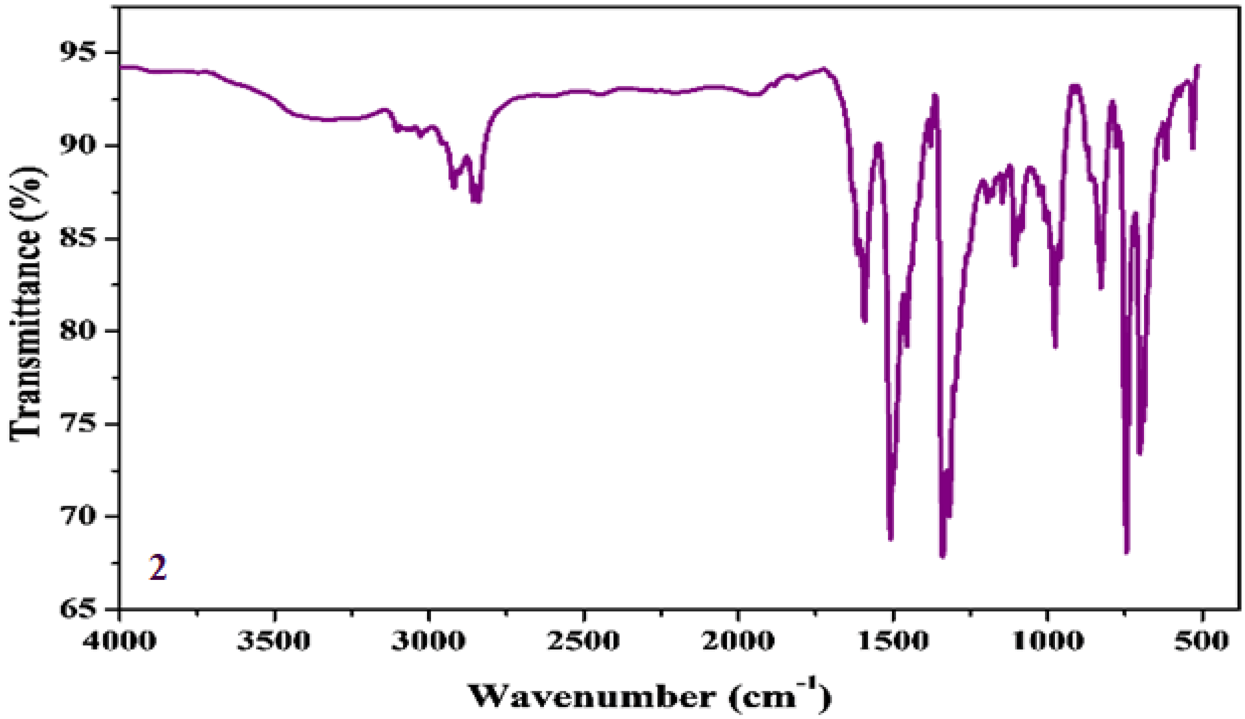

Figure S3: FT-IR spectrum of 2

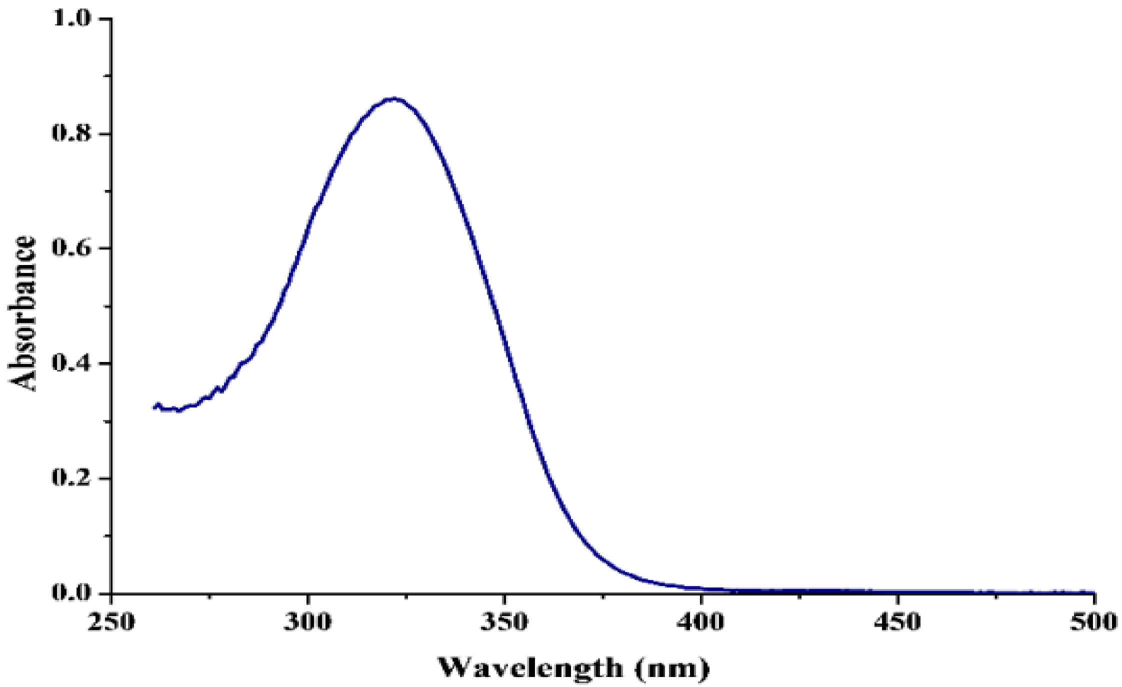

Figure S3: UV-VIS spectrum of 1 in Chloroform

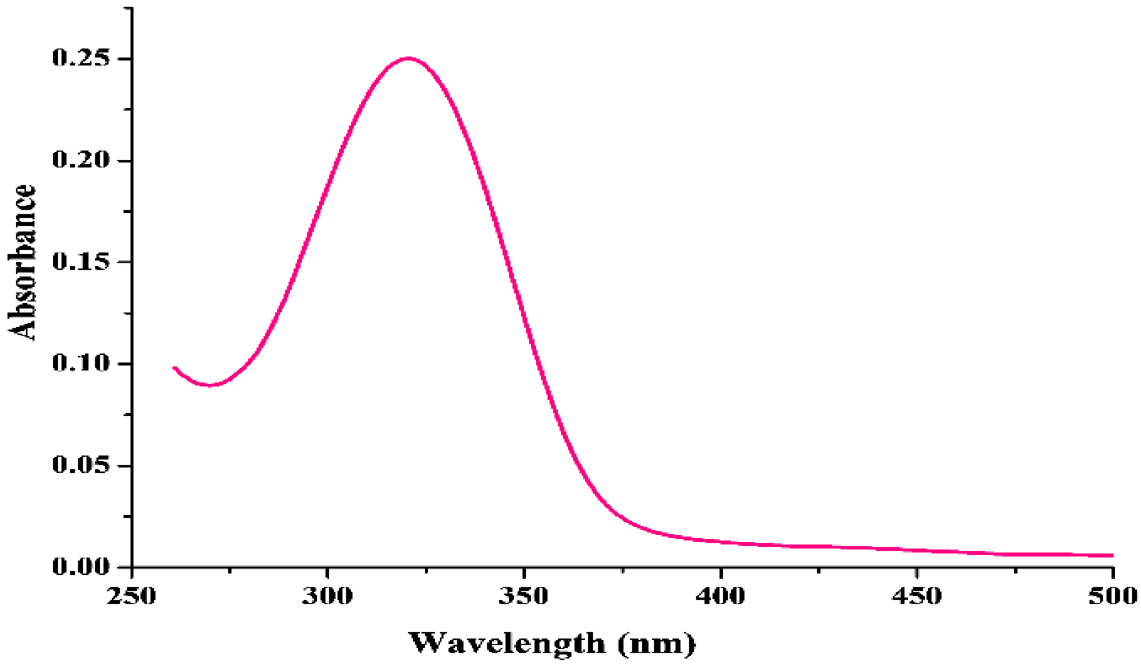

Figure S4: UV-VIS spectra of 2 in Chloroform

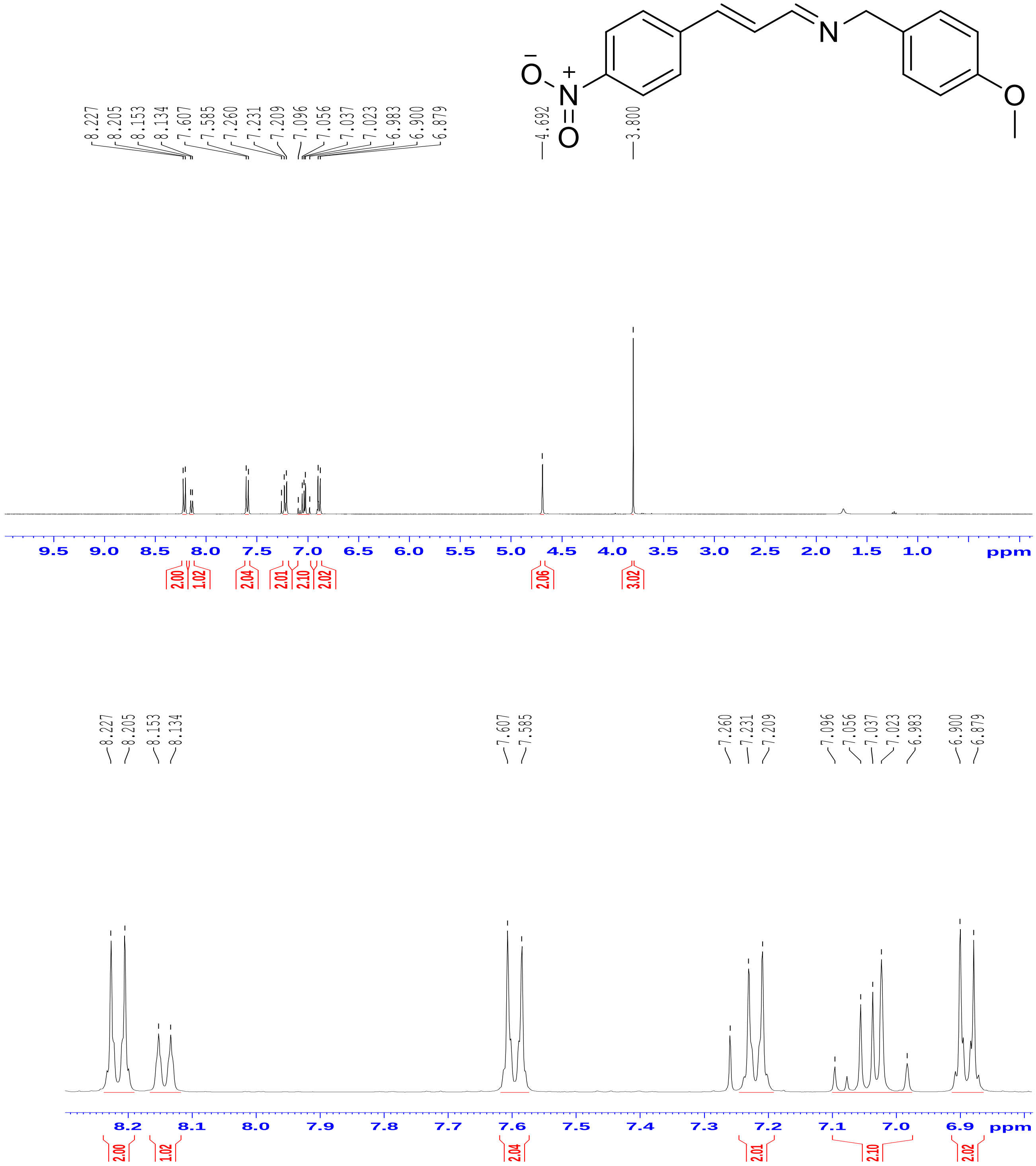

Figure S6: ^{1}H NMR spectrum of 1

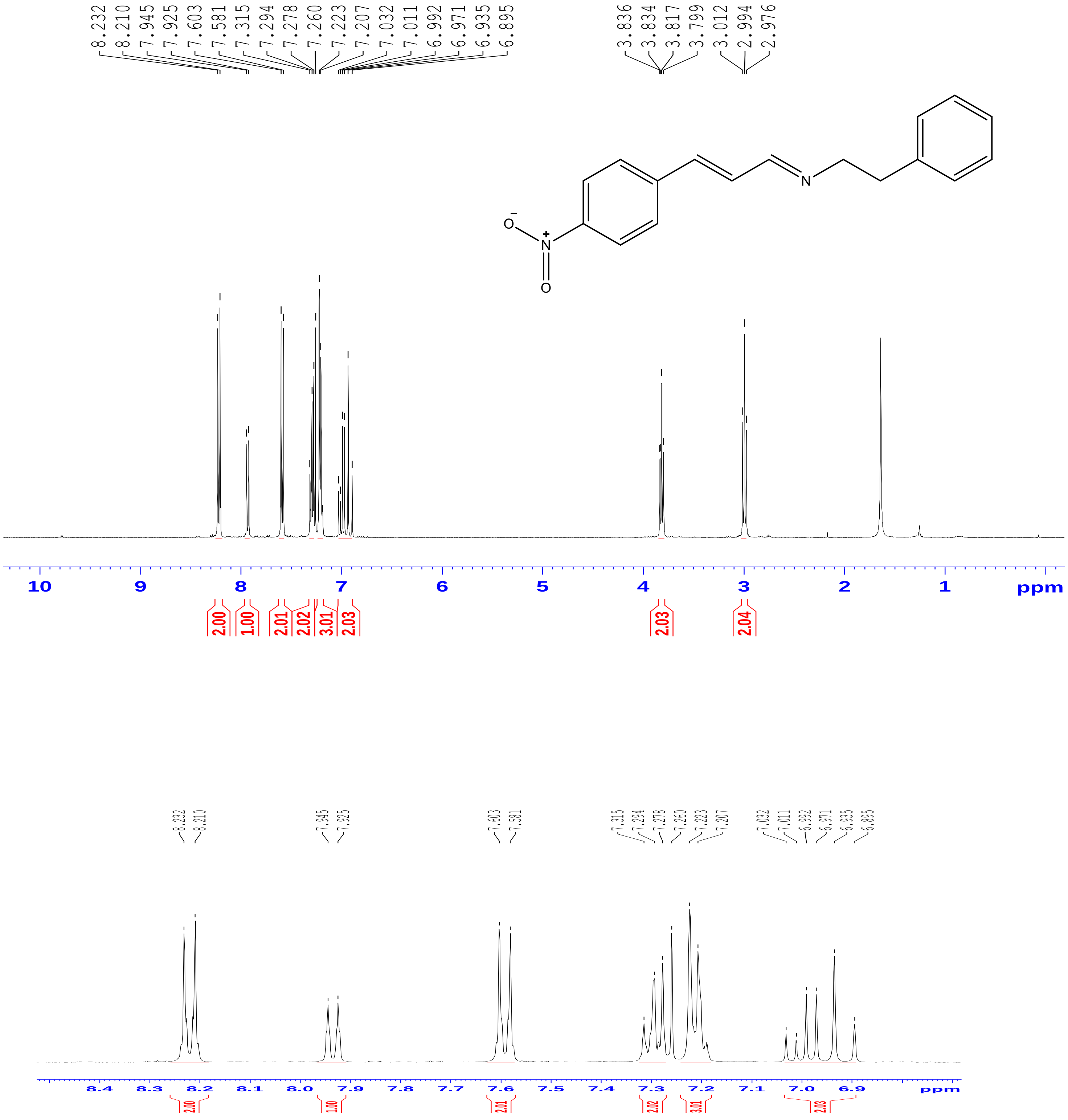

Figure S7: ^{1}H NMR spectrum of 2

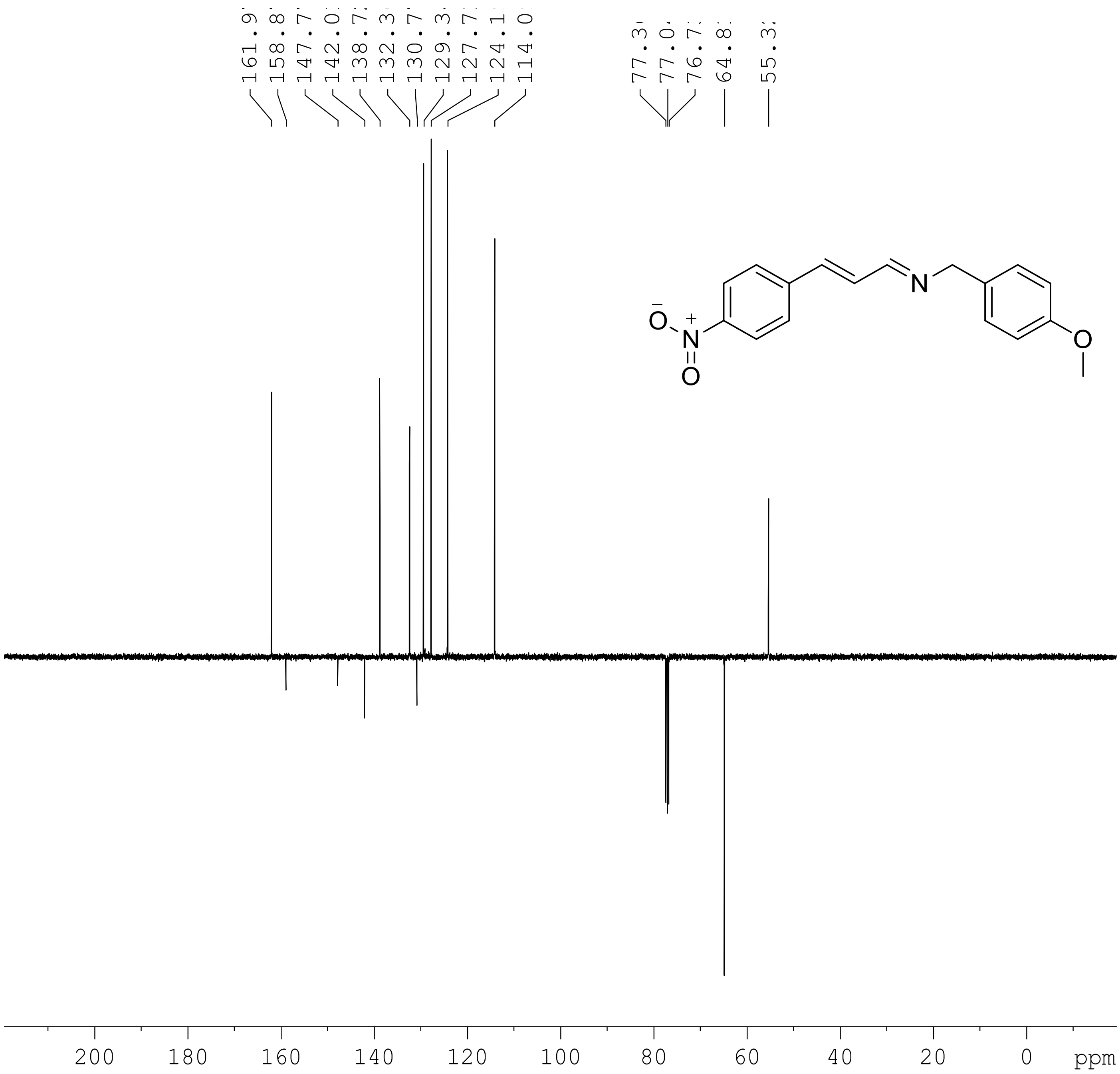

Figure S8: ^{13}C NMR spectrum of 1

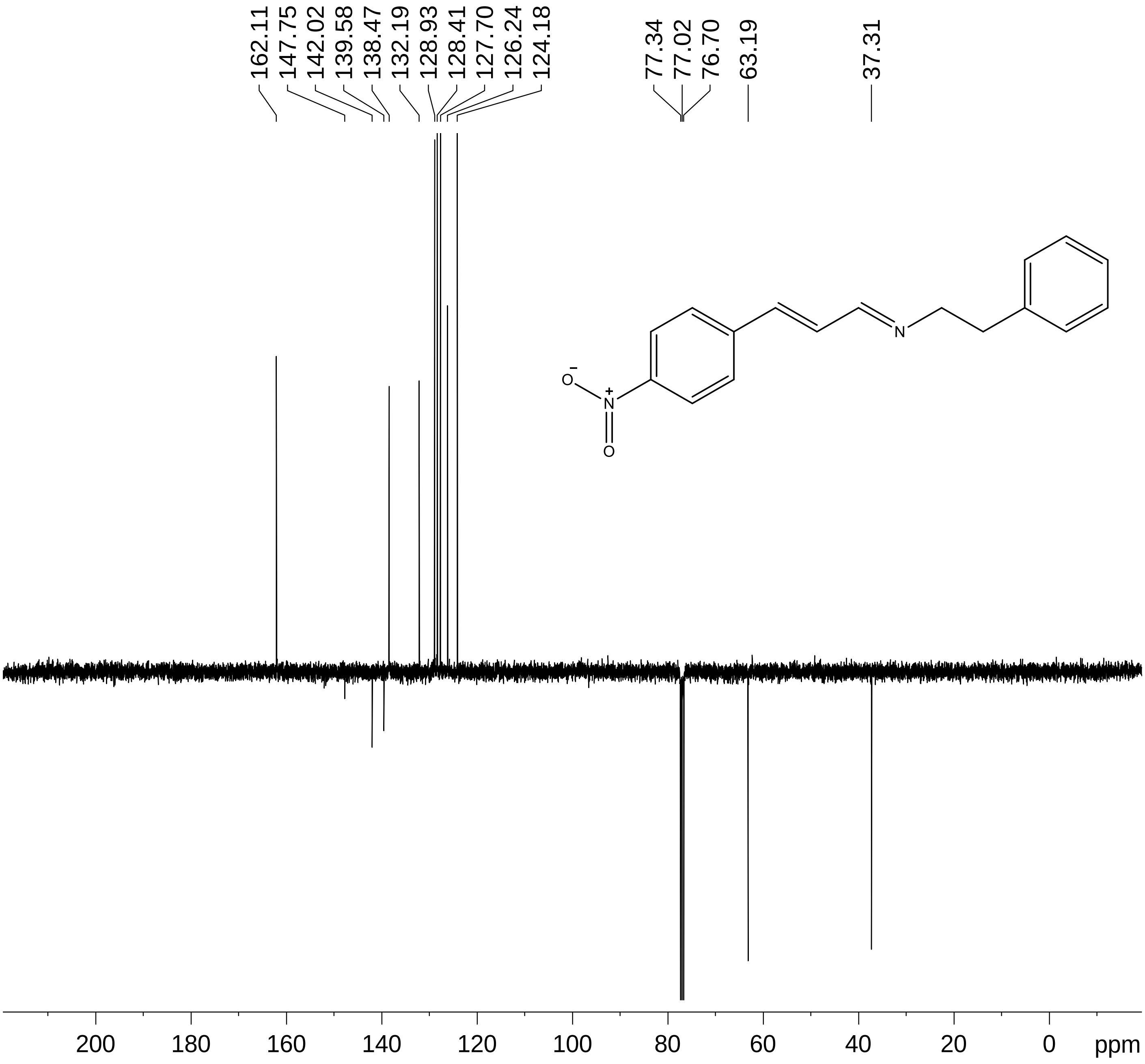

Figure S9: ^{13}C NMR spectrum of 2

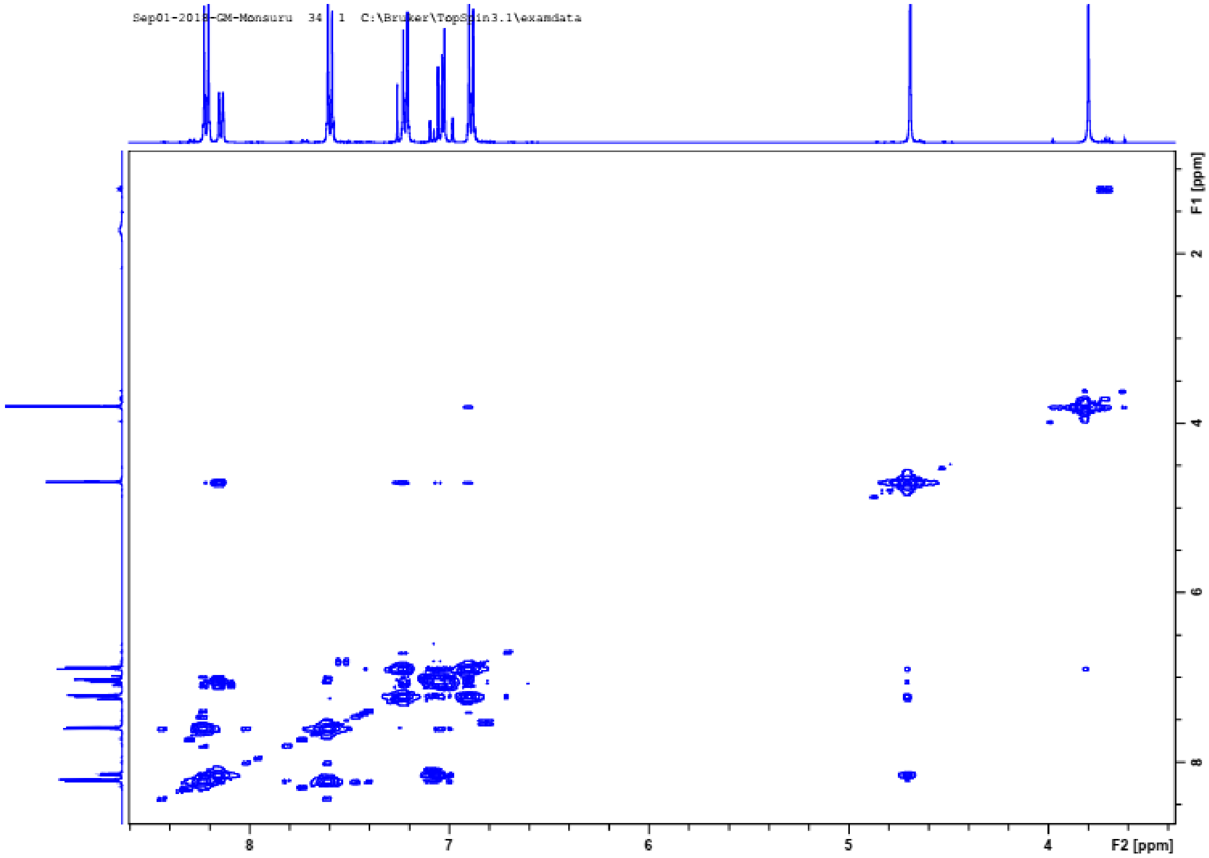

COSY spectrum of **1**

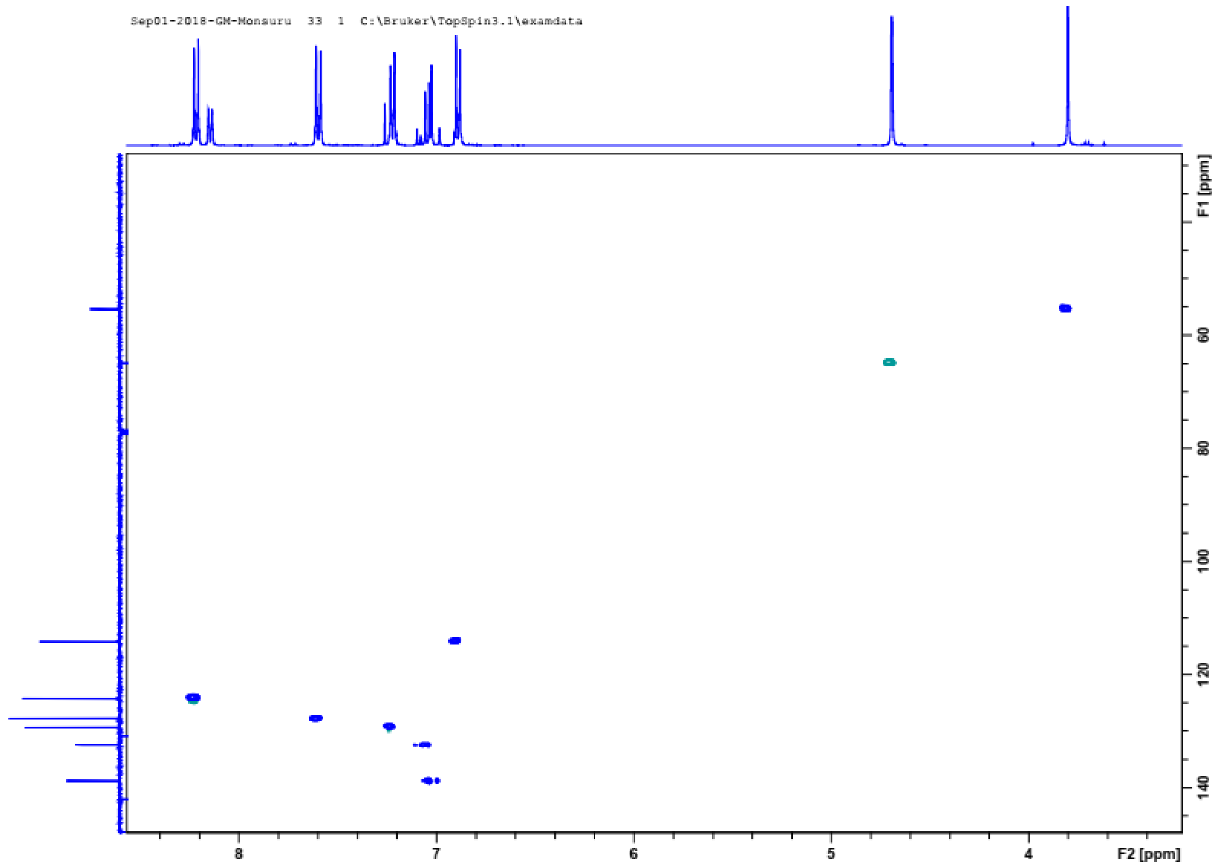

HSQC spectrum of **1**

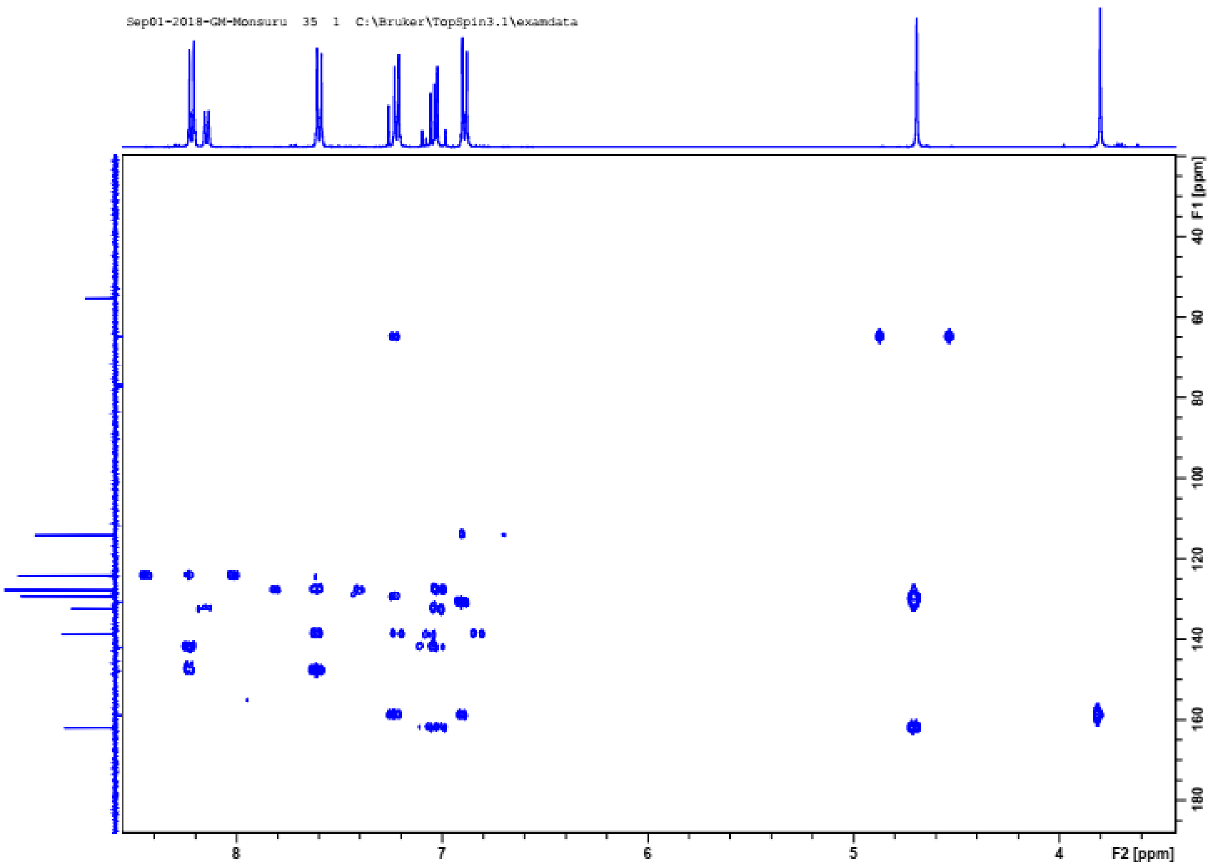

HMBC spectrum of **1**

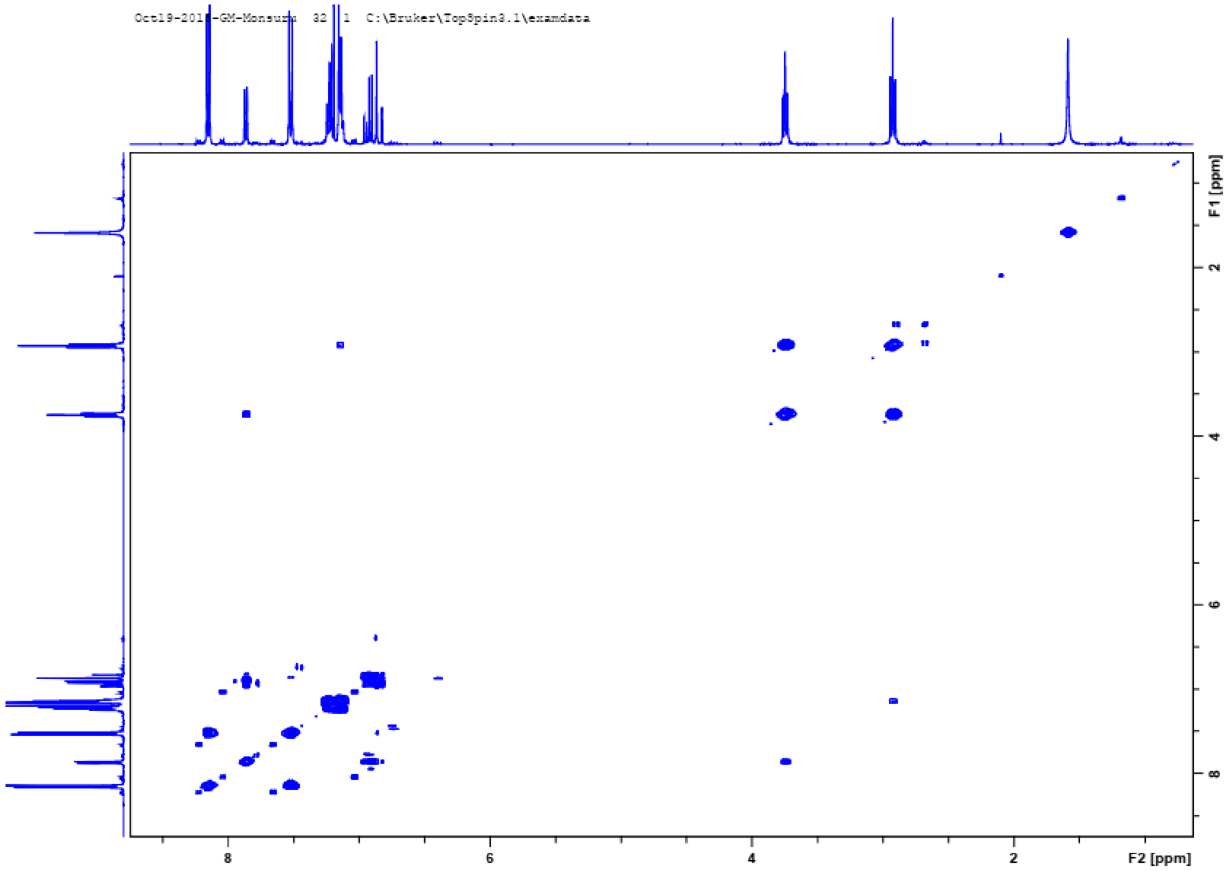

COSY spectrum of **2**

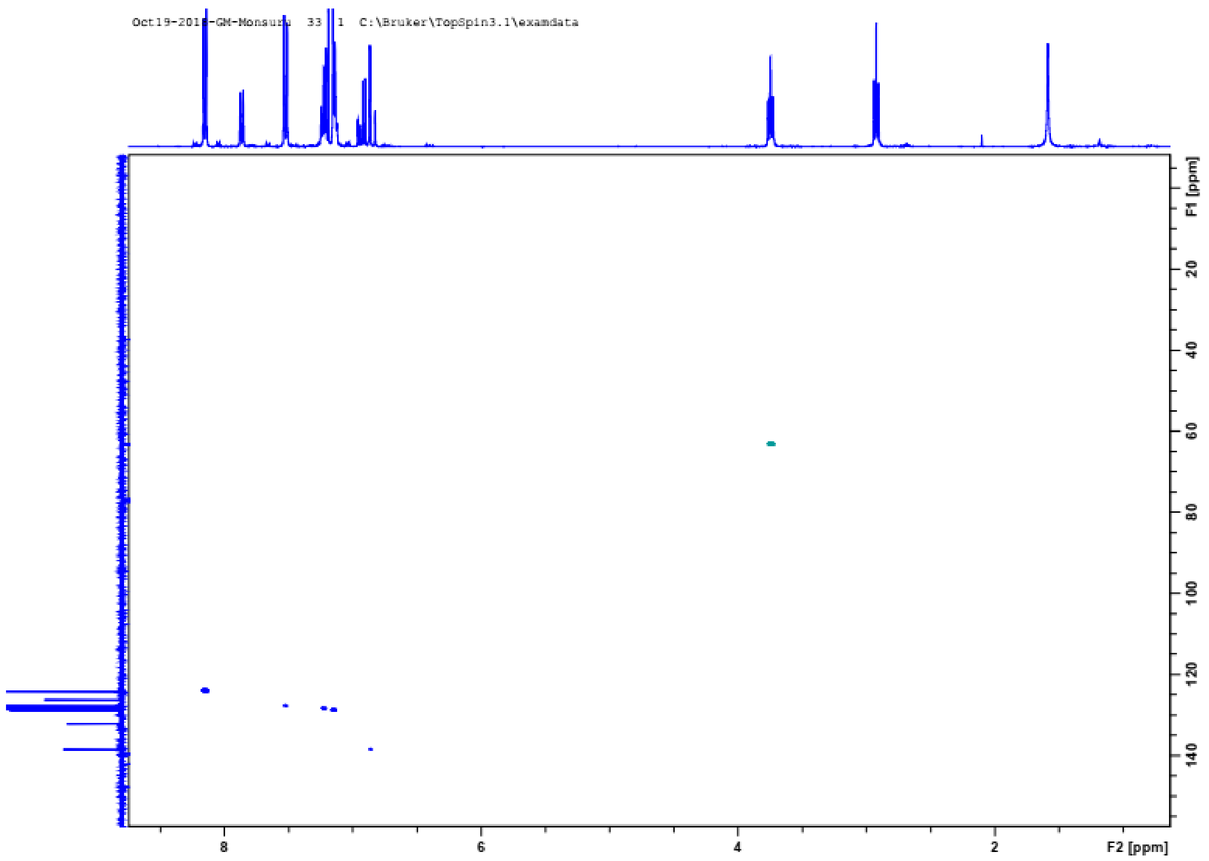

HSQC spectrum of **2**

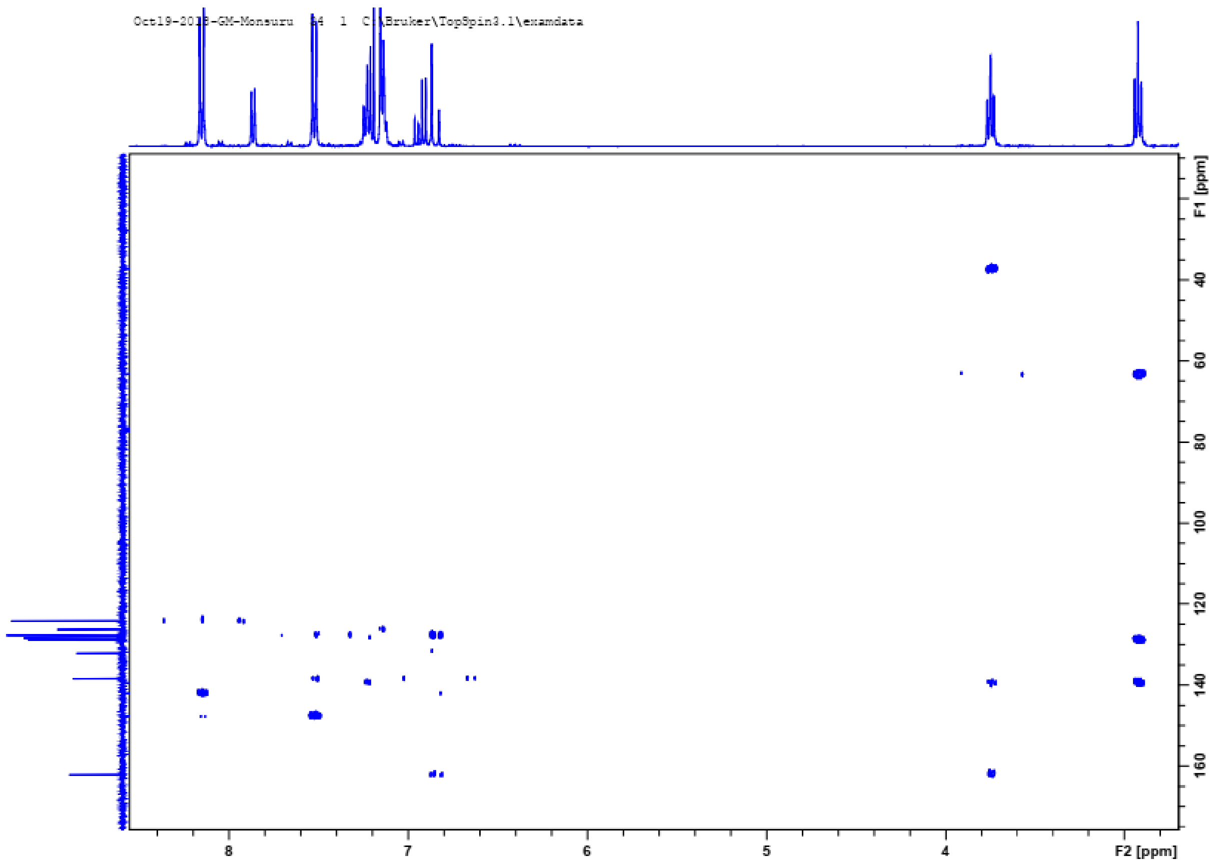

HMBC spectrum of **2**

Figure S10: 2D spectra of compounds 1 and 2

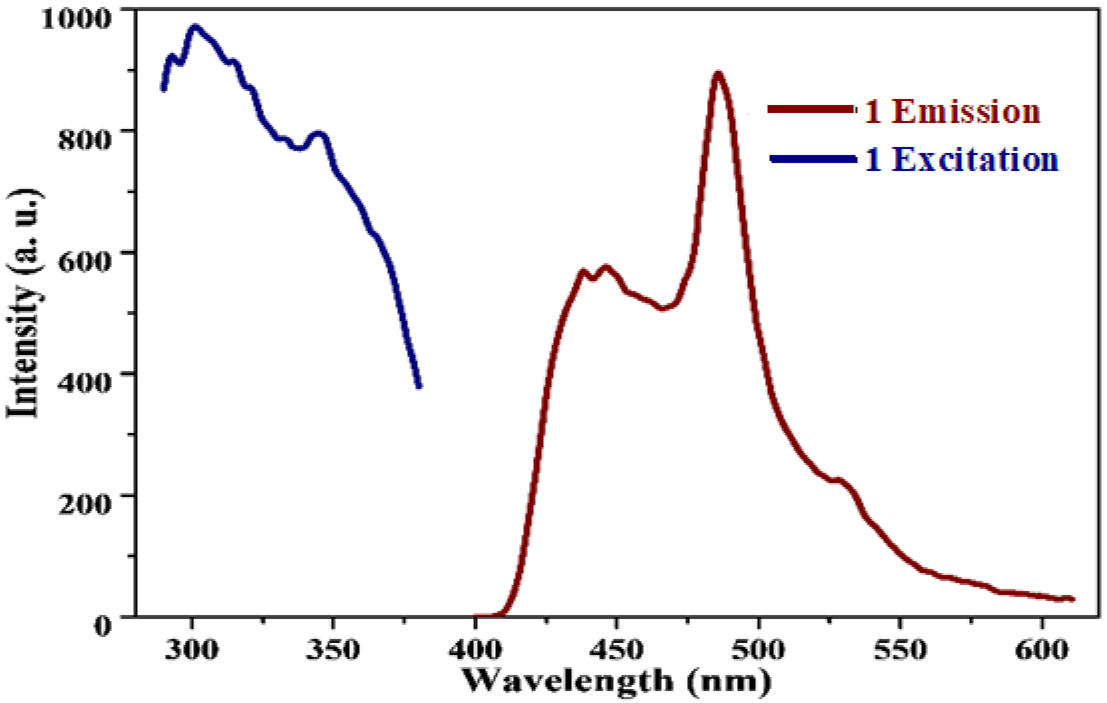

Figure S11: Photoluminescence excitation and emission spectrum of 1

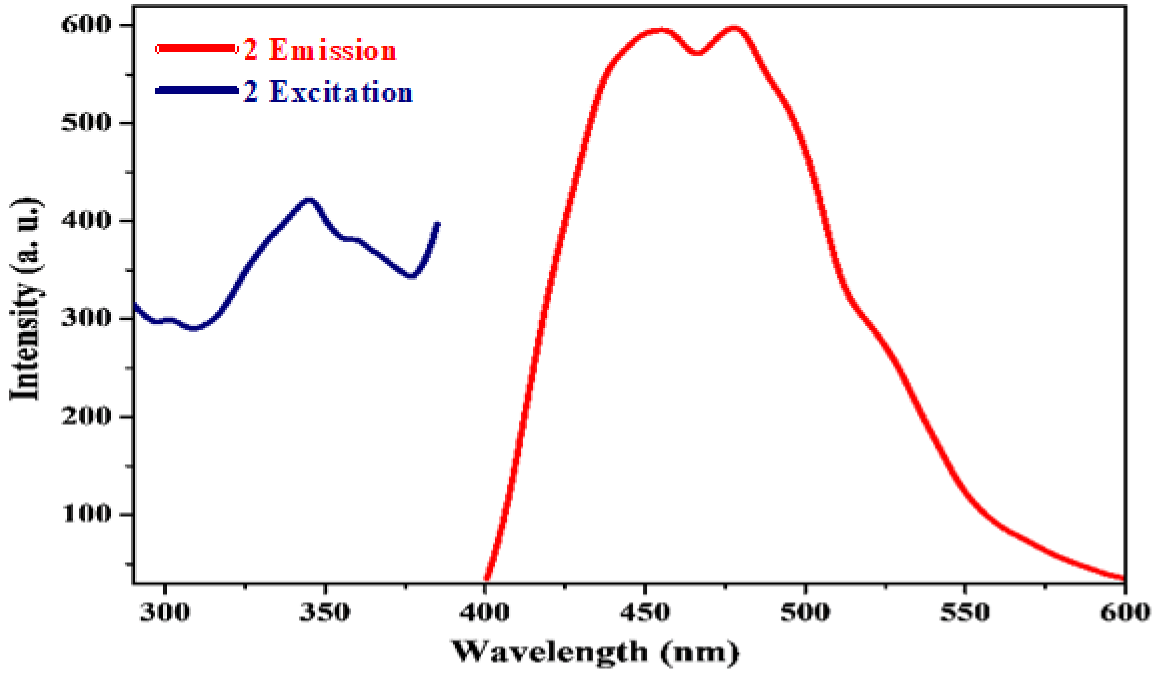

Figure S12: Photoluminescence excitation and emission spectrum of 2

Table S1: ^{1}H and ^{13}C NMR chemical shifts of the Schiff base compounds **1** and **2**

**1**				**2**			
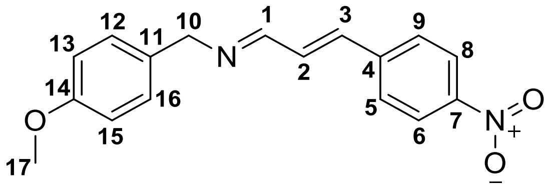				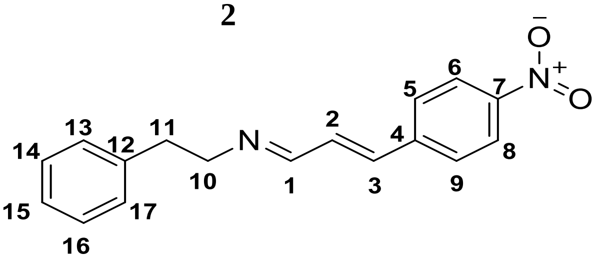			
**Atom**	**δ $^{1}H^{a,b}$**	***J* (Hz)**	**δ $^{1}C^{a,b}$**	**Atom**	**δ $^{1}H^{a,b}$**	***J* (Hz)**	**δ $^{13}C^{a,b}$**
**1**	8.14	7.60	161.97	**1**	7.94	8.20	162.11
**2**	7.06	7.60, 16.10	132.36	**2**	7.00	8.20, 16.10	132.19
**3**	7.00	16.10	138.77	**3**	6.98	16.10	138.47
**4**	-	-	142.00	**4**	-	-	142.02
**5/9**	7.59	8.80	127.71	**5/9**	7.59	8.80	127.70
**6/8**	8.21	8.80	124.19	**6/8**	8.22	8.80	124.18
**7**	-	-	147.77	**7**	-	-	147.75
**10**	4.69	-	64.81	**10**	3.82	7.40	63.19
**11**	-	-	130.77	**11**	2.99	7.40	37.31
**12/16**	7.21	8.6	129.34	**12**	-	-	139.58
**13/15**	6.89	8.6	114.09	**13/17**	7.21-7.32	c	128.93
**14**	-	-	158.87	**14/16**	7.21-7.32	c	126.24/128.41
**17**	3.79	-	55.32	**15**	7.21-7.32	c	126.24/128.41

[a]Solvent ($CDCl_3$), [b]400 MHz for ^{1}H and 100 MHz for ^{13}C, [c]The coupling constants could not be determined due to overlapping of signals.

Table S2: Calculated dipole moments (μ), polarizability (α) and the first hyperpolarizability (β) components (a.u.) for the Schiff base compounds

Compound	μ_x	μ_y	μ_z
1	-3.78	1.42	-1.62
2	3.41	0.89	0.44

Compound	α_{xx}	α_{yy}	α_{zz}	α_{xy}	α_{xz}	α_{yz}
1	-168.66	-129.39	-127.23	5.73	-5.81	-3.74
2	-170.66	-123.64	-116.22	-7.20	-11.58	2.77

Compound	β_{xxx}	β_{yyy}	β_{zzz}	β_{xyy}	β_{xxy}	β_{xxz}	β_{xzz}	β_{yzz}	β_{yyz}	β_{xyz}
1	-727.40	-51.38	-19.32	13.92	-15.92	-178.26	-26.52	-12.26	-13.91	-22.33
2	258.35	-38.72	17.26	-36.68	24.60	36.91	17.59	-4.67	16.34	-1.86

Table S3. Thermodynamics parameters of 1 and 2

Molecules	ZPE ($kJmol^{-1}$)	ΔH^0 (au)	C_v ($Jmol^{-1}$)	ΔS^0 ($Jmol^{-1}$)	G^0 (au)
1	788.29	-992.20	310.44	527.95	-992.26
2	779.54	-916.99	290.90	505.81	-917.05

^{1}H and ^{13}C NMR spectroscopic study of compound 2.

In compound **2**, Two signals (integrating to two protons each) at δ 3.82 and δ 2.99, appearing as a triplet each, exhibit COSY interaction with a coupling constant of δ 7.40 Hz, which are attributed to H-10 and H-11, respectively or vice versa. The signal around δ 3.82 shows HMBC correlation to a carbon resonance at δ 162.11. The corresponding proton (δ 7.94) to the carbon at δ 162.11 shows COSY correlation to proton signal around δ 7.00 with a coupling interaction of 8.20 Hz. The proton at δ 7.00 also displays a COSY correlation to a signal at δ 6.98 with a coupling constant value of 16.00 Hz (indicating a *trans*-relationship), hence, the signal at δ 7.00 was assigned to H-

2. Since the proton (δ 7.94) corresponding to the carbon resonance at δ 162.11 shows an HMBC interaction with a signal attributed C-10 or C-11, therefore, this proton (δ 7.94) was assigned to H-1. This assignment confirmed the resonances at δ 3.82 and δ 2.99 to be labelled as H-10 and H-11, respectively. By elimination, the signal at δ 6.98 was assigned to H-3. Both H-2 and H-3 show HMBC correlations to a quaternary carbon at δ 142.02 (assigned to C-4), while only H-3 displays HMBC interaction with a methine carbon signal at δ 127.70. The corresponding proton (δ 7.59) to the carbon registered at δ 127.70 exhibits a COSY correlation to a proton resonance around δ 8.22 with a coupling interaction of 8.80 Hz indicating an *ortho*-relationship. Therefore, the proton signal around δ 7.59 was assigned to H-5/9 and the one recorded at δ 8.22 was assigned to H-6/8 using *ortho*-relationship and elimination. H-6/8 is highly de-shielded by its *ortho*-nitro group. H-11 displays HMBC correlations to a quaternary carbon at δ 139.58 and a methine carbon resonating at δ 128.93, which were assigned to C-12 and C-13/17, respectively. The corresponding proton (δ 7.21-7.32) to C-13/17 was assigned to H-13/17. However, due to overlap of signals around δ 7.21-7.32, H-14/16 and H-15 were not explicitly assigned. All other carbon resonances were assigned and distinguished according to the ^{13}C attached proton test, HSQC and HMBC.
